# A Low Level of High-Density Lipoprotein Cholesterol Predicts All-Cause Mortality Within 30 Days in Hospitalized Elderly Patients

**DOI:** 10.7759/cureus.82805

**Published:** 2025-04-22

**Authors:** Hirofumi Kitamura, Mitsuaki Kameko, Fumio Kunimoto, Kazuhiko Kotani

**Affiliations:** 1 Department of Laboratory Medicine, Tochigi Prefectural Medical and Social Welfare College, Utsunomiya, JPN; 2 Department of Medical Technology, Faculty of Medical Science and Technolgy, Gunma Paz University Graduate School of Health Sciences, Takasaki, JPN; 3 Department of Internal Medicine, Gunma Paz Hospital, Numata, JPN; 4 Division of Community and Family Medicine, Jichi Medical University, Shimotsuke, JPN

**Keywords:** death, hdl, hospitalization, older age, prognosis

## Abstract

Background: Whether low levels of high-density lipoprotein cholesterol (HDL-C) in the blood determine mortality has not been fully elucidated among elderly patients. It is thus a particular concern to see the predictive implication of HDL-C for post-hospitalized mortality in an elderly population.

Methods: This study was planned to investigate the HDL-C level at admission to a hospital in 493 patients of ≥70 years of age. The outcome was death from any cause within the first 30 days after admission. Cox proportional hazards models were used for the analyses. In addition, the time-dependent (follow-up period) receiver operating characteristic (ROC) curve analysis of HDL-C was performed for all-cause mortality.

Results: The median age of patients was 89 years, and the proportion of male patients was 41%. The median HDL-C level at admission was 41 mg/dL. Deaths occurred in 89 patients (18%) during a median follow-up period of 27 days. In a multivariate model, the HDL-C showed a hazard ratio of 0.977 (95% confidence interval (CI) 0.955-0.998). The results of the ROC curve analysis on all-cause mortality demonstrated that the area under the curve value was 0.67 (95% CI 0.60-0.74) and the cut-off value of HDL-C was 31 mg/dL.

Conclusions: A low HDL-C level at admission was a predictor of all-cause mortality within 30 days in hospitalized elderly patients. The cut-off value of HDL-C was deemed to be low in considering the reference value in daily practice. When elderly patients are admitted, the HDL-C level could be the focus of attention for the prediction of the prognosis and disease management.

## Introduction

A particle of high-density lipoprotein (HDL) has several properties, including anti-inflammation, anti-oxidation, and anti-thrombotic effects and endothelial protection [1-4]. The low level of HDL-cholesterol (HDL-C) is known to be a marker of high mortality in various pathological conditions such as coronary artery disease [5-10]. A significant association has been identified between low HDL-C levels and high all-cause mortality in the general population [10], while recently, extremely high levels of HDL-C have been pointed out to be associated with high all-cause mortality [11-16].

From the viewpoint of biology, aging can reduce HDL functionality, such as delaying HDL maturation, lowering the activity of HDL-related enzymes (e.g., antioxidant paraoxonase), and changing HDL-related apolipoprotein content (e.g., fragmentation of apoA1, an increase in serum amyloid A) [17-22].

An epidemiological study on the elderly population (mean age: 74 years) reported a significant association between low or extremely high HDL-C levels and high all-cause mortality [23], which is in line with the general consensus [10-16]. Therefore, the influence of HDL on mortality is a matter of concern in the elderly; indeed, the findings have been controversial. Another epidemiological study reported that the inverse relationship between HDL-C levels and mortality in elderly patients of 75-99 years of age with mortality was weak in comparison to the younger patients [14]. Notably, several clinical studies that examined the relationship in hospitalized elderly patients reported that a low level of HDL-C might predict all-cause mortality, but that the significance was lost in a multivariate analysis [24-26].

The number of elderly people is rapidly increasing in developed countries, especially Japan [27]. Early discharge and care transition are needed to improve disease management in hospitalized elderly people [28]. Despite this need, few studies have examined the association between biochemical markers and short-term mortality outcomes in elderly hospitalized patients. Therefore, the objective of the present study was to investigate HDL-C levels at admission as a predictor of all-cause mortality within 30 days in hospitalized elderly patients.

## Materials and methods

This retrospective cohort study was conducted at a local hospital (Gunma Paz Hospital, Numata, Japan) between April 2020 and March 2022. Patients of ≥70 years of age (n = 493) who were admitted and discharged from the hospital during this period were included. The outcome was death from any cause within 30 days after admission. Patients with all clinical information for the analysis were included, and patients using lipid-lowering drugs were excluded. This study was approved by the Research Ethics Committee of Gunma Paz University.

Clinical data were collected from the medical records. The data referred to the variables studied in the prior literature on mortality: age, sex [14,15], body mass index (BMI) [29], serum albumin (Alb) [30], triglycerides (TG) [31], low-density lipoprotein cholesterol (LDL-C) [32-34], C-reactive protein (CRP) [35-37], creatinine (Cre) [35-37], blood glucose (Glu) [38], and hemoglobin (Hgb) [39]. In this study, HDL-C levels were measured in the hospital laboratory using an enzymatic homogeneous assay (Sekisui Medical Co., Ltd., Tokyo, Japan). The laboratory data were measured in a non-fasting serum sample.

For the baseline data at admission, continuous data are expressed as the median (interquartile range), and categorical data are expressed as numbers. Predictions of all-cause mortality were statistically analyzed using a univariate analysis (crude analysis) and the Cox proportional hazards model, and all the aforementioned clinical data were used as adjusted variables in the multivariate analysis. In addition, we observed the predictive performance of HDL-C using the receiver operating characteristic (ROC) curve analysis on all-cause mortality. The ROC curve analysis was performed in a time (follow-up period)-dependent manner. The area under the curve (AUC) value and the cut-off value of HDL-C for all-cause mortality were examined by the ROC curve analysis. Stat-Flex software program (version 6; Artec Co., Ltd., Osaka, Japan) for statistical analyses and EZR software program (Saitama Medical Center, Jichi Medical University, Saitama, Japan) for the time-dependent ROC curve analysis were used. A p-value of <0.05 was considered to indicate statistical significance.

## Results

The clinical data of patients at admission (age, sex, BMI, and blood test variables) are summarized in Table 1. The median age of patients was 89 years. The proportion of male patients was 41%. The median HDL-C level at admission was 41 mg/dL (interquartile range: 33-52 mg/dL). During a median follow-up of 27 (interquartile range: 16-30) days, 89 patients (18%) died within 30 days of admission.

**Table 1:** Baseline characteristics of patients BMI, body mass index; Alb, albumin; HDL-C, high-density lipoprotein cholesterol; TG, triglycerides; LDL-C, low-density lipoprotein cholesterol; Cre, creatinine; Glu, blood glucose; CRP, C-reactive protein; Hgb, hemoglobin.

Variable	Median or n	Interquartile range
Age (years)	89.0	84.0-93.0
Gender (male/female)	201/292	
BMI (kg/m^2^)	18.4	16.2-20.8
Alb (g/dL)	3.1	2.6-3.5
HDL-C (mg/dL)	41	33-52
TG (mg/dL)	73	56-99
LDL-C (mg/dL)	91	70-116
Cre (mg/dL)	0.75	0.57-1.07
Glu (mg/dL)	113	97-139
CRP (mg/dL)	2.05	0.38-6.46
Hgb (g/dL)	11.4	10.0-12.5

The crude analysis identified the following predictors of all-cause mortality: male sex (hazard ratio (HR) 1.528, 95% CI 1.009-2.316), low BMI (HR 0.937, 95% CI 0.878-1.000), low Alb (HR 0.303, 95% CI 0.220-0.418), low HDL-C (HR 0.948, 95% CI 0.932-0.966), low LDL-C (HR 0.993, 95% CI 0.986-0.999), high Cre (HR 1.251, 95% CI 1.059-1.478), high CRP (HR 1.053, 95% CI, 1.020-1.086), and low Hgb (HR 0.778, 95% CI 0.703-0.862) (Table 2). Furthermore, a multivariate analysis identified the following predictors of all-cause mortality: low HDL-C (HR 0.977, 95% CI 0.955-0.998), low Alb (HR 0.455, 95% CI 0.286-0.724), and high Cre (HR 1.267, 95% CI 1.045-1.537) (Table 2). The HR of 0.977 in the HDL-C level means that the risk of death decreases by 2.3% for each 1 mg/dL increase in HDL-C. Thus, HDL-C, Alb, and Cre were independent predictors for all-cause mortality. In this analysis, the significance of sex, BMI, LDL-C, CRP, and Hgb was respectively diminished.

**Table 2:** Predictive impacts of each variable on 30-day all-cause mortality HR, hazard ratio; CI, confidence interval; BMI, body mass index; Alb, albumin; HDL-C, high-density lipoprotein cholesterol; TG, triglycerides; LDL-C, low-density lipoprotein cholesterol; Cre, creatinine; Glu, blood glucose; CRP, C-reactive protein; Hgb, hemoglobin. The significance level was set at *p<0.05.

	Univariate model			Multivariate model		
Variable	HR	95%CI	p-value	HR	95%CI	p-value
Age (years)	0.996	0.967-1.026	0.782	1.016	0.983-1.051	0.342
Male	1.528	1.009-2.316	0.045*	1.130	0.704-1.813	0.614
BMI (kg/m^2^)	0.937	0.878-1.000	0.050*	0.946	0.876-1.021	0.155
Alb (g/dL)	0.303	0.220-0.418	<0.001*	0.455	0.286-0.724	0.001*
HDL-C (mg/dL)	0.948	0.932-0.966	<0.001*	0.977	0.955-0.998	0.035*
TG (mg/dL)	0.999	0.993-1.004	0.643	0.999	0.993-1.009	0.800
LDL-C (mg/dL)	0.993	0.986-0.999	0.033*	1.003	0.997-1.009	0.383
Cre (mg/dL)	1.251	1.059-1.478	0.009*	1.267	1.045-1.537	0.016*
Glu (mg/dL)	1.000	0.996-1.005	0.840	1.001	0.997-1.005	0.592
CRP (mg/dL)	1.053	1.020-1.086	0.001*	1.015	0.980-1.052	0.404
Hgb (g/dL)	0.778	0.703-0.862	<0.001*	0.901	0.799-1.016	0.090

**Figure 1:**
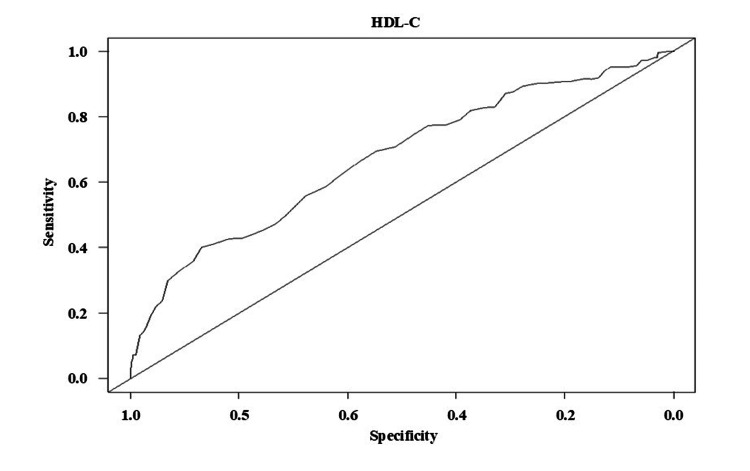
The time-dependent ROC curve analysis for HDL-C at 30 days on all-cause mortality The threshold was estimated by Youden’s index. AUC value = 0.67 (95%CI 0.60-0.74), sensitivity = 0.40, specificity = 0.87, cut-off value = 31 mg/dL. ROC, receiver operating characteristic; HDL-C, high-density lipoprotein cholesterol

The results of the time-dependent ROC curve analysis on all-cause mortality demonstrated an AUC value of 0.67 (95% CI 0.60-0.74, p < 0.05) (Figure 1). The cut-off value of HDL-C for all-cause mortality was 31 mg/dL.

## Discussion

In the present study, a low HDL-C level at admission predicted all-cause mortality within 30 days in hospitalized elderly patients. This suggests that HDL-C could be important for predicting the prognosis and disease management with a comparatively shorter observation period when elderly patients are admitted to the hospital. Since HDL-C is an easily measured standardized marker, this study's findings would be useful for daily practice.

To date, the clinical significance of HDL-C in all-cause mortality remains ambiguous across prior studies [24-26]. The weaker significance of HDL-C has been found in the multivariate analyses in particular [24-26]. The difference in the study methods might have influenced the results; for instance, in addition to the study populations, prior studies had a follow-up period of >1 year (approximately 1.5 years [24], four years [24], 590 days [26]), unlike the present study. Such a longer observation period used in prior studies could have weakened the influence of HDL-C on mortality due to various confounders that arise over a longer time. Thus, the present study is relevant to demonstrate the independent significance of the HDL-C level on all-cause mortality, even using the 'multivariate analysis' and to reveal that the predictive value of HDL-C in mortality may be effective for outcome in a 'shorter time' after admission.

In this work, we attempted to evaluate the predictive ability of HDL-C. From the results of the ROC curve analysis demonstrating the significant predictive performance of HDL-C, its cut-off value was estimated to be 31 mg/dL. The reference levels of HDL-C in daily practice were over 40 mg/dL [40]. A comparatively low level of HDL-C could thus be suggestive of the all-cause mortality in this population. Suggesting such a cut-off value would be clinically helpful, while we remain careful when using the cut-off value, as the AUC value was not always high.

The bio-etiology by which a low HDL-C level predicted death was not clearly determined in the present study. Aging is recognized to be involved in a reduction in HDL functionality, but the function of HDL in protecting against disease progression does not appear to be completely lost in the elderly [17-22]. Thus, patients with low HDL levels at admission can exhibit HDL dysfunction, possibly leading to death. In general, HDL is indicated to be catabolized immediately and/or consumed in patients with severe diseases, which reduces the HDL-C level [6,7]. Additionally, HDL synthesis is reduced during the period of disease progression [41,42]. Therefore, the low HDL-C level at admission is reflective of a highly progressive disease condition, which can be associated with death. As the HDL-C level may not directly mean HDL functionality, further investigation is required for these assumptions.

In this study, the low Alb and high Cre levels were shown to predict mortality independently. These findings are in line with prior knowledge, namely, Alb is a marker of the nutritional and inflammatory status [43], and low Alb is reported to increase the risk of all-cause mortality in hospitalized elderly patients [44-47]. Acute kidney injury and/or decline in renal function, as expressed by high Cre, are also reported to increase the mortality in elderly people [48,49].

The present study had several limitations. This was a retrospective observational study. It was conducted in a single hospital. Although all the variables of markers that were selected for the outcomes considering the prior literature on mortality [29-37] were not always significant in this study, this may be affected by study settings (i.e., a difference in study designs, patient populations, or hospitals). The elderly patients were often hospitalized with complex conditions (e.g., multiple diseases, unknown causes of physical status); accordingly, information on diseases for administration and death was not fully obtained. The relationship among the HDL-C, mortality, and follow-up period is of additional interest, although it is out of the scope of the present study. These limitations should be addressed in future research.

## Conclusions

In summary, in addition to low Alb and high Cre levels, a low HDL-C level at admission was shown to be a predictor of 30-day mortality in hospitalized elderly patients. The cut-off value of HDL-C was deemed to be low considering the reference value in daily practice. When elderly patients are admitted, the HDL-C level may predict the prognosis and could be useful for disease management. The present study had limitations; therefore, further studies are warranted.
